# A remarkable benefit of group psychotherapies with gender dysphoric adults among a case study

**DOI:** 10.1192/j.eurpsy.2021.1461

**Published:** 2021-08-13

**Authors:** H.N. Can, A. Polat

**Affiliations:** 1 Psychiatry, Kocaeli University, Kocaeli, Turkey; 2 Department Of Psychiatry, Kocaeli University, Kocaeli, Turkey

**Keywords:** gender dysphoria, group psychotherapy

## Abstract

**Introduction:**

A person who has gender dysphoria can experience some difficulties in his/her/their life (as we can see even in language). These difficulties are mostly because of the binary gender system. Especially in countries like Turkey, homophobia is very common. Because of that, a homosexual person can think that he/she/they is a transgender.

**Objectives:**

At this point, group psychotherapies are worthwhile, especially for differantial diagnosis. These individuals can share their experiences about the process. On this way, they can also explore themselves.

**Methods:**

We have a monthly group psychotherapy for transgender and gender nonconforming adults in our clinic. We accept the individuals who have problems about gender identity. All trans adults who apply to us and are considered to be suitable are directed to the support group.

**Results:**

An individual, aged 20, who defines herself as a FtM trans has joined to the group for 4-5 months. She was in hurry and had decided to go under surgery for gender transition. She was referred to surgery by another clinic which didn’t have a group psychotherapy. So she applied to our clinic. During the group psychotherapy, she realized that she was homosexual and gived up gender transition.

**Conclusions:**

Group psychotherapy is helpful for trans individuals to reduce psychological distress and manage their process. This study shows that it is not the only benefit of group support psychotherapy. It also helps the individuals to explore themselves, like our case. Its benefit can be as major as saving themselves from an irreversible step and its consequences.

